# Fabrication of Textile-Based Dry Electrode and Analysis of Its Surface EMG Signal for Applying Smart Wear

**DOI:** 10.3390/polym14173641

**Published:** 2022-09-02

**Authors:** Hyelim Kim, Soohyeon Rho, Sora Han, Daeyoung Lim, Wonyoung Jeong

**Affiliations:** 1 Material and Component Convergence R&D Department, Korea Institute of Industrial Technology (KITECH), Ansan 15588, Korea; 2 Department of Nano Science and Technology, Sungkyunkwan University, Suwon 16419, Korea

**Keywords:** textile electrode, conductive sheet, embroidery, knitting, surface electromyography

## Abstract

Ag/AgCl hydrogel electrodes, which are wet electrodes, are generally used to acquire bio-signals non-invasively. Research concerning dry electrodes is ongoing due to the following limitations of wet electrodes: (1) skin irritation and disease when attached for a long time; (2) poor adhesion due to sweat; and (3) considerable cost due to disposable use. Accordingly, electrodes in film, embroidery, and knit forms were manufactured from conductive sheets and conductive yarns, which are typical textile-type dry electrode materials, using different manufacturing methods and conditions. The prepared electrodes were conducted to measure the morphology, surface resistance, skin-electrode impedance, EMG signal acquisition, and analysis. The conductive sheet type electrode exhibited a similar skin-impedance, noise, and muscle activation signal amplitude to the Ag/AgCl gel electrode due to the excellent adhesion and shape stabilization. Embroidery electrodes were manufactured based on two-dimension lock stitch (Em_LS) and three-dimension moss-stitch (Em_MS). More stable EMG signal acquisition than Em_LS was possible when manufactured with Em_MS. The knit electrode was manufactured with the typical structures of plain, purl, and interlock. Although it was possible to acquire EMG signals, considerable noise was generated as the shape and size of the electrodes were changed due to the stretch characteristics of the knit structure. Finally, the applicability of the textile-type dry electrode was confirmed by combining it with a wearable device. More stable and accurate EMG signal acquirement will be possible through more precise parameter control in the future.

## 1. Introduction

Electromyography (EMG) is a biological electric potential generated when a muscle is activated; it is the technique for recording and evaluating the electric signals related to muscle activity (myoelectric). EMG is typically measured using invasive and non-invasive methods. When measuring the EMG by an invasive method, it is measured directly inside or near the muscle by inserting an electrode [1]. On the other hand, for the EMG measured by a non-invasive method, it is measured on the skin surface. Accordingly, the surface electromyography (sEMG) can be measured more easily and safely for the user than the invasive method. Therefore, the non-invasive method is preferred over the invasive method [1,2]. A commonly used electrode is a silver/silver chloride (Ag/AgCl) type hydrogel electrode, which is a “wet electrode” used to measure high-precision signals. The EMG signal is considered an alternating current signal, and it is affected the skin-electrode impedance. The wet electrode is good at attaching to the skin. Thus, it is known that it reduces the electrical impedance between the skin and the electrode to a smaller value than that of a “dry electrode”. Wet electrodes are mainly used by directly attaching to the skin of a patient in the medical field. However, since this is a disposable, there are issues regarding attachment and cost when applied to devices used in sports, fitness, or daily life. In addition, there are problems, such as skin irritation that occurs when used for a long time, non-reusability, poor adhesion when attaching clothes, and discomfort during operation. Therefore, many commercially available sEMG for wearable devices and fiber-based dry electrodes are being continuously developed to replace them [3].

Research on wearable technology and devices for improving health care, fitness, and convenience has been active owing to the increasing interest and demand for smart wear. In particular, smart wear for collecting bio-signals is worn mainly in close contact with the human body, so electrodes or sensors for measuring it are being developed as an in-cloth embedded in clothes [4,5]. Accordingly, studies on dry electrodes based on soft materials using conductive yarns, fabrics, and films are being conducted. Electrodes to which this soft material is applied are manufactured by applying a conductive sheet [6], embroidery [7], and knitting [8] since they are simple, scalable, and highly customizable. Since the human body has a curved shape, there is room for detachment due to operation when Ag/AgCl hydrogel electrode is attached. However, if the electrode is made in the form of in-cloth, it can be more stable to acquire bio-signal in movement since it is in close contact with the human body. Thus, since accurate signal collection is essential for adhesion and body movement to use the sEMG electrode embedded in clothing, research to develop a suitable textile electrode is also underway.

Studies have been conducted on EMG acquisition according to the size and shape, dynamic conditions, or clothing pressure to fabricate conductive sheet and screen printing-based textile electrodes [6,9,10]. Spanu et al. (2021) fabricated stretchable textile electrodes using a screen printing process with PEDOT:PSS and verified the signal quality under dynamic conditions [9]. They reported that the textile electrodes could be more accurate than conventional disposable Ag/AgCl electrodes. Kim et al. (2020) prepared textile electrodes for each electrode size using a commercial conductive sheet to develop a textile electrode for applications to smart clothing. They analyzed the EMG performance according to the clothing pressure [6]. It was reported that the optimal electrode size was 20 mm in diameter, and the contact area and SNR increased with decreasing impedance as the clothing pressure was increased. 

The embroidery technique is also a representative method for manufacturing textile-type electrodes and can be used to manufacture various types of electrodes according to designs using conductive yarn on fabric [11,12]. Depending on the embroidery technique, it can be produced as a 2D type lock stitch or 3D type moss stitch (pile stitch). Generally, a lock stitch is used, but the moss stitch is used to improve the touch and contact area when touching the skin. Research is currently ongoing [7,13]. Goncu-Berk and Tuna (2021) analyzed the EMG signal quality between embroidered textile-based EMG electrodes and the skin. Embroidered textile-based EMG electrodes were integrated onto the sleeves of custom-drafted t-shirts with a set-in and raglan sleeve [11]. As a result, the set-in sleeve pattern style performs better and when the fit is more close-fitting, but the EMG signal quality declines significantly in loose conditions. Kim et al. (2022) developed an embroidery electrode with excellent EMG acquisition while improving economic efficiency by reducing the amount of conductive yarn. The circle-type and wave-type electrodes were analyzed according to density [12]. Accordingly, the wave type was excellent, and it was confirmed that the EMG performance of the sample filled with the full electrode and the sample filled with three lines were similar. Lee et al. (2020) fabricated a fabric vest socket with pile-type embroidered electrodes for a high-level upper amputee [7]. The SNR of the pile-type embroidered electrode showed similar performance to the conventional electrode, and the most comfortable device could be manufactured by combining the combining flexible fabric electrode. 

The knitted form has the advantage of adhering to a curved body and skin due to its elasticity, and it has the advantage of producing a seamless piece of clothing by inserting electrodes into the garment at one time using a knitting machine [8]. Accordingly, studies on knitted electrodes for biosignal collection are continuously being conducted [9,10,14,15]. Euler et al. (2021) prepared six knitted electrodes by size, construction, topology, and pressures and analyzed and compared their contact impedance [9]. The contact surface with the skin became wider as the electrode size increased. In addition, the surface roughened, and the impedance decreased.

Therefore, in this study, a representative textile-based dry electrode was manufactured according to the manufacturing process and conditions using a commercialized conductive sheet and conductive yarn to replace the disposable electrode with low impedance and high accuracy. The morphology, skin-electrode contact impedance, and sEMG acquisition were analyzed. Based on this, an attempt was made to confirm the applicability of the textile-type electrode in smart wear in the future.

## 2. Experimental

### 2.1. Materials

Three types of textile-based dry electrodes were prepared according to the manufacturing process. To manufactured sheet-type dry electrode, two types of commercial conductive sheets in development were used which are composed of silver and carbon paste with different tensile properties (Figure S1). The conductive sheet with about 730% elongation was obtained from the ‘T’ company. It has a thickness of about 0.3 mm, it has a structure in which the surface in direct contact with the skin is made of a carbon paste layer, and a silver paste layer is positioned below it. In addition, the conductive sheet having an elongation of about 25% was obtained by the ‘S’ company. It has a thickness of about 0.4 mm, silver nanowire and carbon nanoplate are used, and the manufacturing form is the same as the above product. For manufacturing embroidery-type and knit-type dry electrodes, the conductive yarn of AMANN group (Silver-tech, AMANN group, Bönnigheim, Germany) was used, which is the same material used in previous study [12]. It has a resistivity of 530 Ω/m. For the conductive sheet and embroidery electrode, commercial polyester-based elastic fabric for application as sportswear was used as the substrate fabric [12]. The substrate fabric was composed of 88% Polyester and 12% Spandex, and the weight and thickness were 451.5 g/m^2^ and 1.0 mm, respectively. The substrate fabric used was a double weave consisting of wale × course of 128 × 88/5 cm. The face of the fabric was composed of flat knitted fabrics, and the back side is double-side knitted fabrics. The designed embroidered and knitted electrodes were manufactured using a technical embroidery machine (SGVA 0109-825, ZSK Stickmaschinen GmbH, Krefeld, Germany) with No. 14 with 90 Nm needle and flat knitting machine (ADF830 24W (12 gauge), STOLL, Obertshausen, Germany).

### 2.2. Sample Preparation

Preparation of Leg Sleeves Embedded the Textile-Based Dry Electrodes

Table 1 shows the sample codes and images of each textile-based electrode. Based on the results of a previous study [6], the diameter of the textile-based dry electrode, according to the manufacturing method, was designed and fabricated in a circular shape of 20 mm.

**Figure 1:**
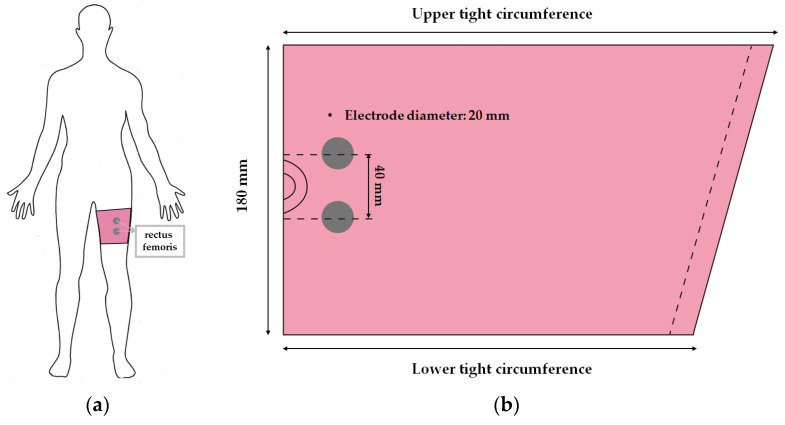
Scheme of (**a**) rectus femoris, which is muscle activation position, and (**b**) fabrication of leg sleeves with embedded textile-based dry electrodes.

The leg sleeves with textile-based dry electrodes embedded were used since the muscle activation in the rectus femoris muscle was chosen in a previous study [6,12]. Accordingly, a pair of electrodes was selected in the same method as in the previous study [12] so that they were located at the corresponding positions. This study was perform on a subject which is same as previous study [12] for sEMG measurements (sex = female, age = 32, height = 175 cm, body weight = 60 kg). Thus, according to the subject’s condition, the length was 180 mm, and the circumferences of the upper and lower thighs corresponding to the position were measured. Based on the results of the previous study [6], the pattern reduction rate (PRR) was applied for 30%, considering the elasticity of the substrate fabric (Figure 1).

First, the conductive sheet-based textile electrode (CS) was prepared by cutting it to a diameter of 20 mm. The sEMG signal was measured by placing the adhesive surface on the substrate fabric so that the inter-electrode distance (IED) of two electrodes was 40 mm, and lamination was performed by hot-pressing (Hydraulic hot press, Korea MP Engineering, Bucheon, Korea). At this time, the hot-pressing condition was carried out at 120 °C for 15 s. Subsequently, it was fabricated by connecting a metal snap for EMG signal collection. Second, embroidery electrodes were fabricated with a lock stitch (LS) produced in a 2D form. A moss stitch (MS) was prepared in a 3D form for comparison according to the embroidery technique. The samples were designed using the EPC_win embroidery design program (ZSK Stickmaschinen GmbH, Germany). When manufacturing using the LS technique, the stitch length and distance were set to 2.0 mm and 0.5 mm, respectively, and when fabricating using the MS technique, the loop height and stitch distance were set to 3.0 mm and 0.5 mm, respectively. Third, the knit electrode was knitted into three structures: plain (PL), purl (PU), and interlock (IN), to compare the performance of the electrode according to the structure. Each sample was designed with STOLL M1 Plus 7.0.038 software, and the electrode part was made of 12 gauge, 2 ply of conductive yarn × 1 ply of spandex were integrated into a 2 ply of non-conductive polyester yarn × spandex 1 ply. At this time, to match the electrode size of 20 mm due to the shrinking characteristics of the knitted fabric, the number of loops and the size of the stitches were designed to be different for each structure. 

### 2.3. Characterization

#### 2.3.1. Morphology of the Textile-Based Dry Electrodes

The morphology of the various types of textile-based dry electrode according to the manufacturing method was observed using a multimedia imaging microscope (RH-2000, KEYENCE Co. Ltd., Seongnam, Korea) at ×35 magnifications. To analysis of 3D images, Zeiss Xradia 510 Versa 3D X-ray microscopes (XRM, Carl Zeiss Microscopy Deutschland GmbH, Oberkochen, Germany) was used. The volume of the area was measured to determine the electrode shape that changes when worn due to the inherent elongated of the knit electrode. The measured condition was same as previous study [12], the voltage and power were 50 kV and 4 W, respectively, and the resolution was 22 μm. The number of taken images was 1601 per sample, and the exposure time was five seconds. To confirm the amount of conductive yarn used in the textile electrode using conductive yarn, the embroidery electrode was calculated and compared according to the stitch length, distance, number of stitches, and fabric thickness through the EPC_win embroidery design program (ZSK Stickmaschinen GmbH, Germany) based on previous research [12]. In addition, the knit electrode was compared with the amount of conductive yarn according to the structure based on STOLL M1 Plus 7.0.038 software.

#### 2.3.2. Sheet Resistance of the Textile-Based Dry Electrodes

The electrical characteristics of the textile-based dry electrode according to the manufacturing method were examined using a four-point probe conductivity meter (RSD-IG 4-Probe, DASOLENG., Cheongju, Korea). In the case of a conductive sheet-type textile-type electrode, it was measured with a metal snap since it is used together with a metal snap. All samples were measured 10 times, and the average value was used.

#### 2.3.3. Skin-Electrode Contact Impedance of the Textile-Based Dry Electrodes

The impedance of the electrodes was related directly to the signal-to-noise ratio (SNR). Accordingly, the electrodes must undergo impedance validation that supports efficient EMG acquirement performance. Samples with two textile-based electrodes located in parallel to the leg sleeve were used to compare the skin-electrode contact impedance value according to the method of manufacturing the existing Ag/AgCl electrode and textile electrode. After attaching the Ag/AgCl electrode or wearing a leg sleeve so that the textile-based electrode made of embroidered and knitted textile-based electrode could contact human skin, the reference electrode and conductive sheet can be connected to a metal snap to measure impedance. On the other hand, the embroidery and knit electrodes were difficult to connect to measure the instrument directly. Thus, a disposable Ag/AgCl electrode was attached to the back of the embroidered and knitted electrodes to measure the impedance. The measuring method was the same as previous study [12]. The distance between both electrodes was set at 40 mm, and the impedance was tested using an impedance analyzer (ZIVE P2 ELECTROCHEMICAL WORKSTATION, Won-A tech. Co. Ltd., Seoul, Korea). The skin-electrode contact impedance was measured with AC sinusoidal signal at the 1 to 1000 kHz frequency range. The skin-electrode impedance value at 100 Hz, targeting the peak frequency component for EMG [16,17], was analyzed to compare the electrode type. The test was conducted without pre-treatments of the skin, such as shaving or exfoliation, respectively. All measurements were performed on the same day on the same subject.

#### 2.3.4. sEMG Measurement and Data Processing of the Textile-Based Dry Electrodes

**Figure 2:**
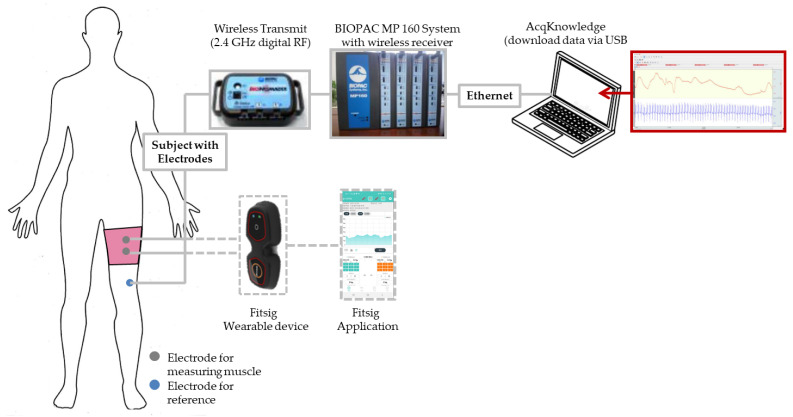
Schematic diagram of the measuring process for the sEMG signal.

Figure 2 shows a scheme of the process for measuring sEMG signal using the biopac and wearable device. The validation of textile electrodes was based on the comparison between EMG signals simultaneously detected, from the same muscle, using the Ag/AgCl hydrogel electrodes and textile electrodes. This study analyzed and evaluated the sEMG signals of the various textile-based electrodes by analyzing the EMG signal characteristics during knee extension. Based on a previous study [6,12], bipolar EMG recordings were obtained using textile-based electrodes on the rectus femoris. 

Before the test, skin preparation was not conducted since no separate skin preparation is required even when wearing smart clothes for electromyography. The location of the corresponding rectus femoris, which is the measurement position, was marked on the skin with a pen. A pair of electrodes was positioned on the midpoint of the muscle for the rectus femoris, and was aligned on the longitudinal axis of the target muscle. A reference electrode (Kendall LTP, Covidien, MA, USA) was placed over the head of the fibula, the electrically neutral bony prominence. The whole data processing of sEMG was conducted using AcqKnowledge 5.0.1 Software (BIOPAC Systems, Inc., Goleta, CA, USA). The recordings were amplified and filtered from 20 to 500 Hz in analog (MP160, BIOPAC Systems, Inc., Goleta, CA, USA). The data were full-wave rectified and averaged with a 100 ms time constant to draw the amplitude of the signals. For the sEMG signal processing, root-mean-square (RMS) operations were performed, and the value was calculated to validate the utility of the sEMG signals. After that, the signal-to-noise (SNR) was calculated using Equation (1):

SNR = RMS_signal_/RMS_noise_ (1)

**Figure 3:**
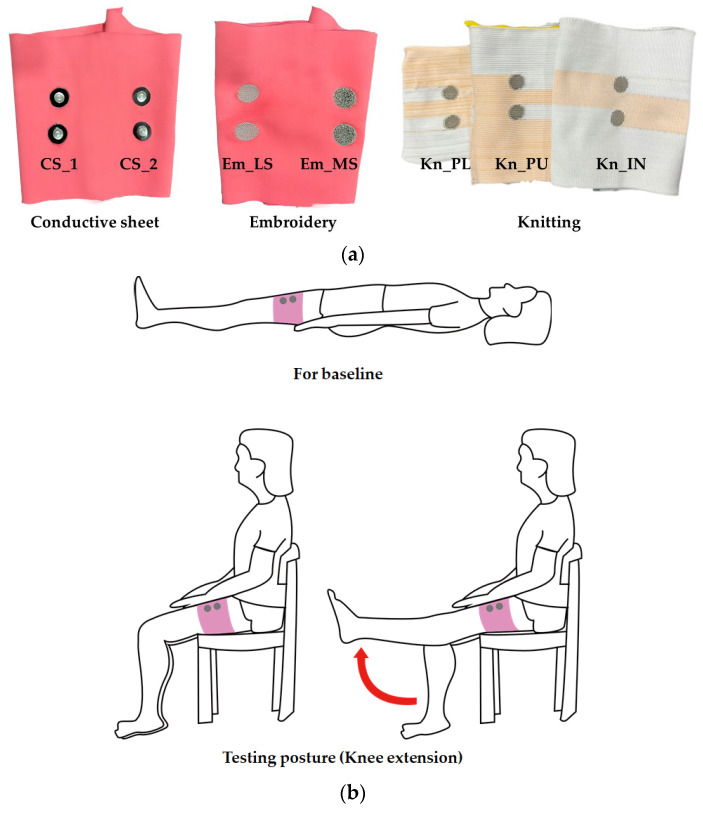
Images for measuring the sEMG signal: (**a**) actual leg sleeve; (**b**) testing posture.

Figure 3 shows a prepared leg sleeve and testing posture for measuring sEMG signal. First, the baseline EMG signals were collected in the supine position for 10 s to measure the baseline electrode noise (Figure 3b). To induce the EMG signal by muscle contraction, with the subject seated, a single-joint knee extension was conducted. The subject extended their knee until the lower leg became parallel to the floor. The test was continuously conducted by five trials for knee extension and flexion. At that time, each phase lasted for five seconds. This study also tested only one subject, the individual difference in muscle activities were not considered. The experiment was conducted at room temperature under 60 %RH. The test action was conducted five times to record the sEMG signal from one subject during intermittent muscle contraction measured. Except the first and last trials, three contractions from five were used to calculate the average activated EMG for comparison.

As shown in Figure 2, to confirm the possibility of using manufactured textile-based dry electrodes for measuring the EMG signals to smart wear, it was measured and confirmed using commercial wearable devices and mobile applications. The commercial wearable device (Fitsig, Roem system, Co. Ltd., Daejeon, Korea) was attached to the textile-type electrode part on the surface of the leg sleeve, and the textile-type electrode on the backside was collected the signal of human skin. The sEMG signal acquirement method was the same as Biopac: three consecutive times were measured. The sEMG signal was compared various types of electrode and the signal obtained from the BIOPAC.

## 3. Results and Discussion

### 3.1. Morphology of the Types of Textile-Based Dry Electrodes

Table 2 lists the morphology and consumption of conductive yarn of the textile-based dry electrode. The surface characteristics of laminating using a conductive sheet, embroidery using conductive yarn, and knit electrode, which are representative dry electrode-manufacturing methods, were compared. First, since the two conductive sheets, CS_1 and CS_2, were manufactured in a film type, they showed a smooth surface without irregularities compared to embroidery or knit electrodes using conductive yarn. In addition, it was confirmed that the conductive sheet-type electrode is significant attached to the fabric without space, it shows the flat surface. Accordingly, the peel strength between the fabric and the electrode made by conductive sheet was about 615 cN/mm, and it was confirmed that it showed better adhesion than the peel strength (440 cN/mm) of the moisture-permeable waterproof fabric for clothing manufactured by the laminating process [18]. At ×35 magnification, a comparison of CS_1 and CS_2 confirmed that the surface of CS_2 has irregularities compared to CS_1. These irregularities are likely related to the degree of skin contact during EMG collection. 

In embroidery electrodes, the lock stitch technique is generally used for durability. On the other hand, as research on textile electrodes progresses, the moss stitch technique has been applied to improve the tactile of the textile electrode in close contact with the skin for collecting biological signals [7]. Accordingly, two representative embroidery techniques were carried out based on the lock stitch (Em_LS) and moss stitch (Em_MS). The Em_LS is produced in 2D form with two conductive yarns, the lower and upper threads, so that the front and back are the same, whereas Em_MS has a single upper thread passing through the fabric to form a loop, so the face side has a pile-shaped 3D structure and the back side shows the same shape as Em_LS. In addition, through the digital image, the Em_MS showed a larger size than the other samples, which was confirmed since the moss stitch in the form of a loop has a spreading shape. When comparing the use of conductive yarn of the embroidery electrodes of the two techniques, 624.0 cm for Em_LS and 878.4 cm for Em_MS, respectively, confirming that the amount of Em_MS used is slightly higher. Since it is manufactured by forming a 3 mm loop, it was confirmed that a large amount of conductive wire is consumed compared to the same area. Nevertheless, when manufactured with smart wear, Em_MS appears to affect the impedance or SNR by increasing the contact area and improving the comfort due to the loop.

In the case of the knit electrode, the unevenness of the surface exposed to the outside is different according to the plain(Kn_PL), purl(Kn_PU), and interlock(Kn_IN) structures. 

Kn_PL was knitted the flattest among the three structures, and the face and back sides were in the wale and course directions, respectively. The loop was knitted most densely within the same magnification. Kn_PU has the same face and back side shape, and it was confirmed that the path inserted in the course direction protrudes compared to the loop in the course direction. In addition, Kn_IN was confirmed in the form, in which the loop in the wale direction protrudes to the face and backside. Accordingly, it was confirmed that the amount of use of the conductive yarn was different, and the Kn_PL, Kn_PU, and Kn_IN were 643.5 cm, 756.5 cm, and 724.5 cm, respectively. Knit electrode could be fabricated of a fabric structure consisting of loops with one row of loops constructed with one continuous yarn and each row of loops connected to the row above and below [8]. Therefore, the shape of the loop being formed according to the knitting structure and the length of the conductive yarn are expected to affect the skin-electrode impedance and EMG acquirement.

### 3.2. Electrical Properties of the Types of Textile-Based Dry Electrodes

#### 3.2.1. Sheet Resistance

**Figure 4:**
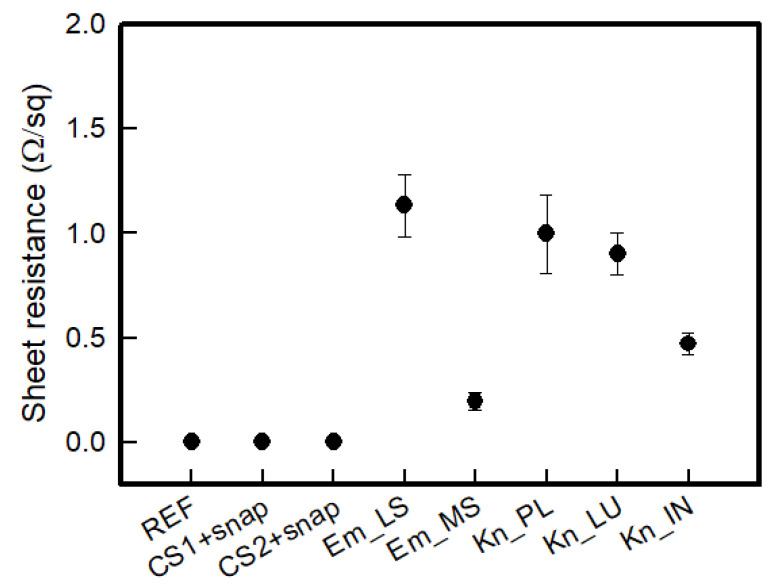
Sheet resistance of textile-based dry electrodes with various types.

Figure 4 shows the sheet resistance to confirm the electrical characteristics according to the manufacturing method and type of the textile-based dry electrode. The sheet resistance was in the order of REF < CS_1 < CS_2 < Em_MS < Kn_IN < Kn_PU < Kn_PL < Em_LS (Figure 4). As mentioned in the experimental method, CS_1 and CS_2 were measured in the same state since snaps are connected and used. Accordingly, the Ag/AgCl electrode was 0.022 ± 0.01 Ω/sq, and CS_1 and CS_2 were 0.004 ± 0.001 Ω/sq and 0.003 ± 0.001 Ω/sq, respectively, indicating lower values than the reference electrode. At the embroidery electrode, the sheet resistance of Em_LS and Em_MS were 1.135 ± 0.148 Ω/sq and 0.197 ± 0.043 Ω/sq, respectively, confirming that Em_MS shows significantly lower values than LS. As confirmed in the previous morphology, the MS manufactured in the loop type has a bulkier shape than the Em_LS manufactured in the stitch type. Thus, the conductive path was enhanced since the connect point in each conductive yarn increases due to the loop. In addition, even in the case of the standard deviation, the Em_MS was less than that of the Em_LS, confirming that the Em_MS could have more stable electrical performance when manufactured. The sheet resistance of the Kn_PL, Kn_PU, and Kn_IN knit structures were 0.997 ± 0.186 Ω/sq, 0.902 ± 0.098 Ω/sq, and 0.472 ± 0.050 Ω/sq, respectively. In the case of knits, as analyzed in the morphology, different results were shown depending on the shape of the loop being formed according to the knitting structure and the length of the conductive yarn. Plain was knitted in the form of one layer, and there were no irregularities, whereas in purl, despite being one layer, it appears that the contact point has increased due to the loop existing in the course direction on both sides. In addition, since there are more contact points between loops in the case of interlock than purl due to loops in the warp direction on both sides, the conductive path was improved, and the sheet resistance was reduced. Accordingly, the increase in the contact point of the conductive material is related to the improvement of electrical performance.

#### 3.2.2. Skin-Electrode Impedance

**Figure 5:**
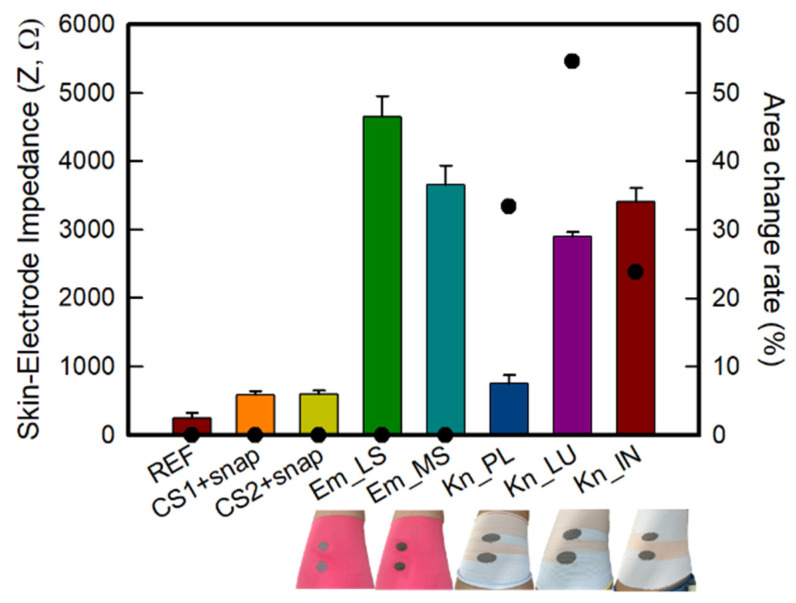
Skin-electrode impedance and area change rate of textile-based dry electrodes with various types.

A low and stable impedance at the skin-electrode interface is key to the high-fidelity acquisition of bio-signals, both acute and long-term [19]. Figure 5 shows the skin-electrode contact impedance of various types of textile-type dry electrodes at 100 Hz. As shown in Figure 5, the skin-electrode contact impedance of the reference electrode was 33.0 ± 1.8 kΩ. Overall, the textile-based dry electrode showed higher values than the reference electrode, and CS_1 and CS_2 were 213.3 ± 121.7 kΩ and 283.6 ± 33.4 kΩ, respectively. The embroidery and knit electrodes made of conductive yarn showed significantly higher values than the conductive sheet. Em_LS and Em_MS were 1851.8 ± 127.2 kΩ and 1570.0 ± 220.9 kΩ, respectively, which was different from the low sheet resistance result of the knit electrode. On the other hand, when only the embroidery electrode was compared, the impedance value of Em_MS was reduced compared to that of Em_LS, similar to the sheet resistance. In the case of the knit electrode, the skin-electrode impedance values of Kn_PL, Kn_PU, and Kn_IN were 401.3 ± 58.5 kΩ, 707.4 ± 95.1 kΩ, and 1989.6 ± 802.3 kΩ, respectively, which was confirmed to be opposite to the sheet resistance. Accordingly, it was confirmed that the impedance of the textile-type dry electrode increased in the order of REF < CS_1 < CS_2 < Kn_PL < Kn_PU < Kn_IN < Em_MS < Em_LS. 

In the case of the previously analyzed sheet resistance, the same electrode size was measured without the leg sleeve being worn. In contrast, in the case of skin-electrode contact impedance, the subject wore a leg sleeve embedded with each electrode from a different manufacturing method and then measured. Accordingly, different results were obtained depending on the shape of the electrode and the contact area that was deformed after wearing [14]. In the case of the conductive sheet, a stable skin-electrode contact impedance was achieved due to its relatively high adhesion as it was manufactured in a flat shape without irregularities compared to the conductive yarn. On the other hand, in the case of Em_LS, the value was larger than that of other dry electrodes. It was relatively difficult to adhere to the skin completely since the fabric exhibited some shrinkage due to its high density during sample production. As a result, the contact area was lower than that of other samples. Hence, the impedance results are also affected. Since Em_MS has a loop shape, as mentioned in the sheet resistance result, the impedance value decreased since the area that can be contacted increases when the bulky shape comes into contact with the skin than the stitch type Em_LS. The knit electrodes showed an opposite trend to the sheet resistance, which appears to have been influenced by the change in the shape of the electrode and the increase in size due to the shear characteristics since the loop changes according to the structure and elasticity, which are inherent characteristics of knits [14]. To confirm the structural change of knitted electrode, Figure S2 was indicated the structural change before and after tensile test of Kn_PL among the three knit structures. As shown in Figure S2, after 80% elongated, it was confirmed that the stain change rates in the wale and course directions of the loop were −1.18% and 18.1%, respectively. Thus, it is expected to affect to EMG signal collection. In addition, it was possible to adhere to the curved side of the body due to the flexibility and bending modulus of the knit. Accordingly, the flattest plain structure exhibits a similar skin-impedance to the conductive sheet since the area that can contact the skin is improved compared to the other two samples. Therefore, the skin-electrode contact impedance decreased as the area with the increased electrode contact to the skin and adhesion. In addition, the conductive sheet and the textile-based dry electrode of the plain knit type could have similar impedance to that of Ag/AgCl.

### 3.3. sEMG Signal and Average Rectified sEMG of the Types of Textile-Based Dry Electrodes

**Figure 6:**
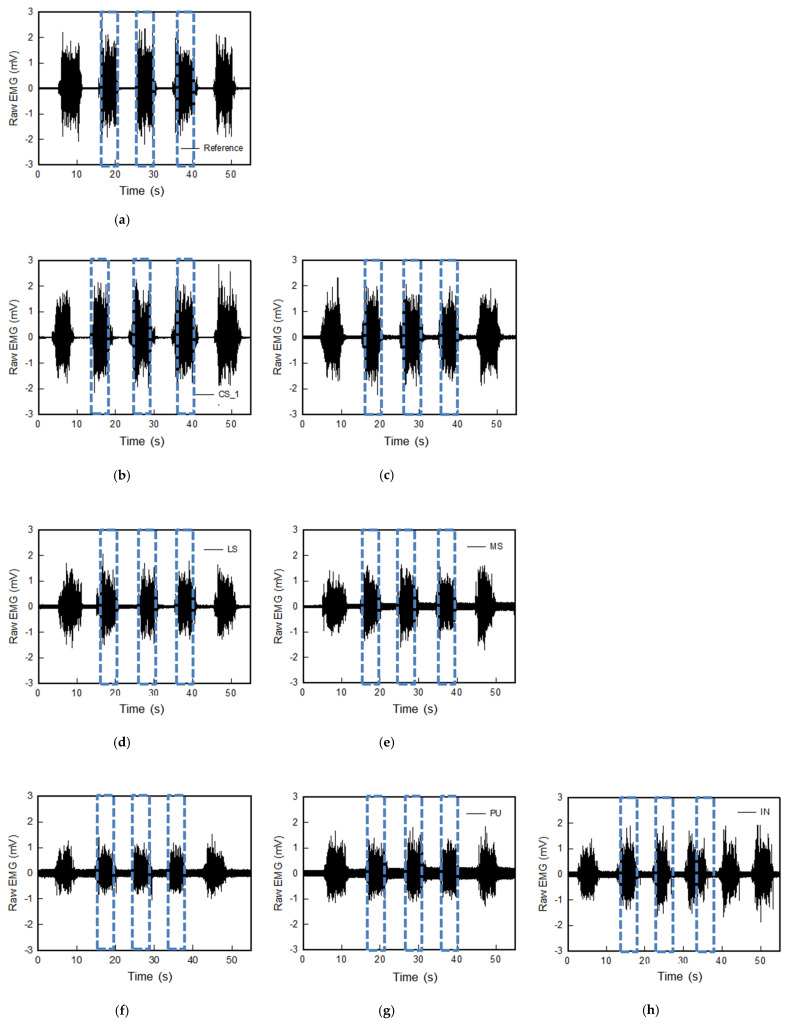
Graph of raw sEMG signals obtained via various electrodes; (**a**) reference, (**b**) CS_1, (**c**) CS_2, (**d**) Em_LS, (**e**) Em_MS, (**f**) Kn_PL, (**g**) Kn_PU, and (**h**) Kn_IN, respectively.

Figure 6 represents the raw sEMG signals of the various textile-based dry electrodes. The raw sEMG signal was intended to confirm the noise of each textile-based dry electrode and the magnitude of the signal obtained during muscle construction. EMG was collected five times by one subject. As shown in Figure 6, including Ag/AgCl electrode, all textile-based dry electrodes were confirmed to be capable of measuring signals during muscle activation. In addition, the noise signal which is supine position of all electrodes were near 0. Nevertheless, each sample showed different noise signal values. When looking at each electrode, the noise of the reference electrode was ± 0.04 mV and the signal acquisition during muscle activation was ± 2.83 mV (Figure 6a). Among the textile-based dry electrodes, the raw EMG signals of CS_1 and CS_2, which are conductive sheets, were similar to the reference. The noise of CS_1 and CS_2 were ± 0.02 mV and ± 0.05 mV, respectively, and signal amplitude during muscle activation were ± 2.85 mV and ± 2.41 mV, respectively. Accordingly, it was found that the textile-based electrode of the conductive sheet type that is in close contact with the skin has sEMG signal acquisition performance similar to that of the Ag/AgCl hydrogel electrode. In addition, the embroidered and knitted electrodes made of conductive yarn showed increased noise and amplitude compared to the conductive sheet electrodes. 

In the case of the embroidery electrode and the knit electrode, different results were shown depending on the manufactured shape and knitting structure. The embroidery electrodes confirmed that Em_MS and Em_LS were ± 2.07 mV and ± 1.98 mV, respectively, which were similar signal acquisition sizes during muscle activation (albeit with a difference in noise). Overall, the knit electrode had larger noise than the embroidery electrode, and the noise was reduced when it was an interlock structure. In the signal amplitude during muscle activation, Kn_PL < Kn_PU < Kn_IN. The shape and structure of the loop, which changes depending on the knitting type during stretching due to elasticity and flexibility, were different [8]. 

**Figure 7:**
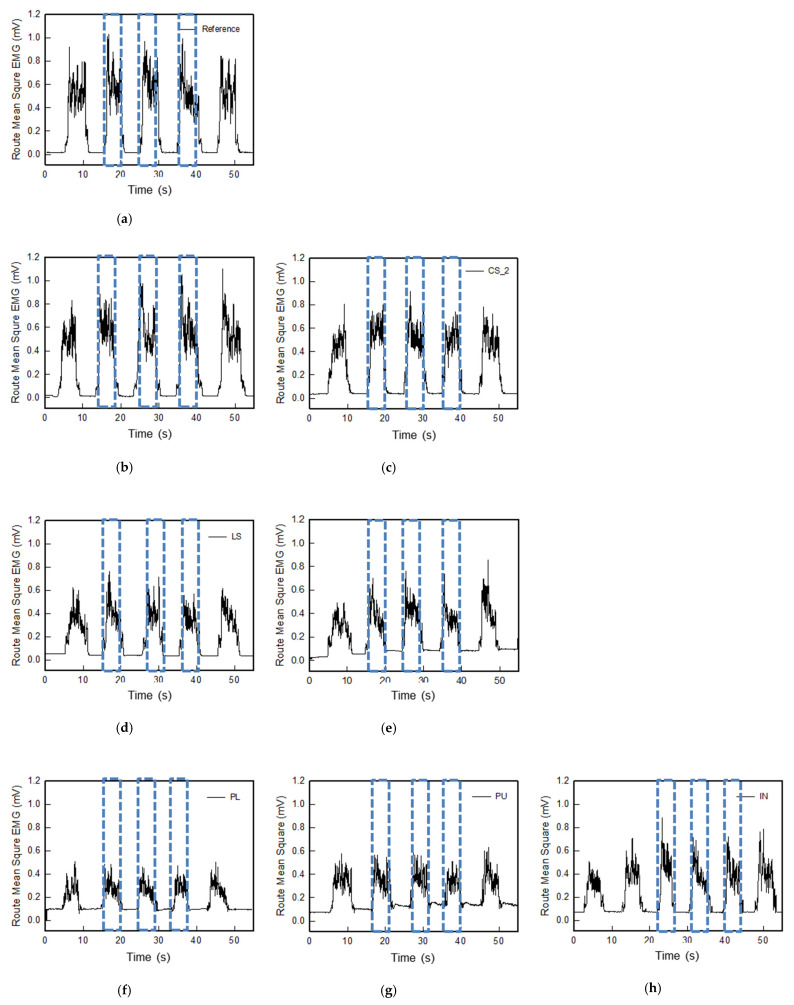
Graph of filtered (20–500 Hz) in analog and full-wave of sEMG signals obtained via various electrodes; (**a**) reference, (**b**) CS_1, (**c**) CS_2, (**d**) Em_LS, (**e**) Em_MS, (**f**) Kn_PL, (**g**) Kn_PU, and (**h**) Kn_IN, respectively.

Figure 7 shows the sEMG signal analysis results for each electrode after filtering raw data and converting it to root mean square (RMS). The baseline noise and muscle activation of the reference electrode and textile-based dry electrodes were compared through RMS EMG signal analysis. In the case of baseline noise, the reference electrode was 0.017 mV, it is similar to zero. The conductive sheet type electrodes CS_1 and CS_2 were 0.016 mV and 0.025 mV, respectively, those were more stable than the reference electrode. As previously confirmed in the raw EMG signal, the baseline noise of the embroidered and knitted electrodes was relatively high, the noise of RMS value of Em_LS, Em_MS is twice higher than Kn_PL, Kn_PU, and Kn_IN. On the other hand, it showed different results depending on the manufacturing method and knitting structure, and showed an opposite trend to some skin-electrode contact impedance results. Em_LS showed higher baseline noise than Em_MS and was the same even after five muscle contraction movements. In the case of Em_MS, the initial baseline showed lower and more stable noise than the reference electrode, but it increased as the muscle contraction motion progressed. When measuring the impedance, it proceeded in a static motion and was less affected by noise, but the EMG signal was measured while muscle contraction motion was being performed. Thus, the noise was affected by movement. Therefore, the conductors in the 3D type Em_MS manufactured with loops, rather than the 2D type Em_LS manufactured with stitches, can move along with the muscle contraction motion, confirming that they were affected.

As shown in Figure 7f–h, the baseline noise of three types of knit electrodes was higher than that of both embroidered electrodes. In addition, the EMG signal appeared differently in the order of Kn_PL < Kn_PU < Kn_IN from the result of skin-electrode contact impedance during muscular activation. In relation to the morphology, the degree of irregularities in the three tissues was observed in the order of Plain < Purl < Interlock. As mentioned above, in the case of skin-electrode contact impedance, the flattest plain as measured in a static state shows a low value and is stable. On the other hand, when the leg sleeve made of the plain structure was worn, the electrode showed a form that expanded in the lateral direction and contracted in the longitudinal direction (Figure S2). Accordingly, as the lateral length of the electrode becomes longer than that of the rectus femoris to be measured, the contact area of the electrode for sensing the corresponding muscle decreases, and sensing adjacent muscles appears to show baseline noise and lower active EMG signal amplitude [6,20]. The purl and interlock structures were similarly expanded in the lateral and longitudinal directions, confirming that the baseline noise was reduced and the active EMG signal amplitude increased since the adjacent muscles were relatively less affected (Figure 5).

**Figure 8:**
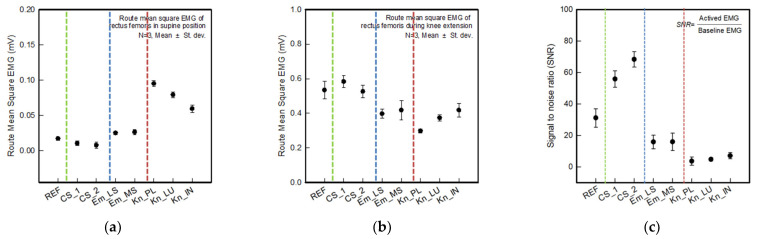
Average rectified sEMG of the baseline during muscle activation for different electrode types. (**a**) Baseline electrode noise; (**b**) sEMG amplitude during muscle contractions exerted by knee extension; (**c**) signal to noise ratio (SNR).

Based on the results of raw EMG and filtered RMS EMG confirmed earlier, Figure 8a,b shows the average rectified sEMG of the baseline and muscle activation for different electrode types. Figure 8a,b show the calculated mean and standard deviation of the three acquired signals out of five (excluding the first and last signal). As shown in Figure 8a, the baseline noise of CS_1 and CS_2 was near 0.00 mV, which is lower and more stable than the commercial electrode. Em_LS and Em_MS showed between 0.01 and 0.02 mV and similar baseline noise compared to the reference electrode. On the other hand, three knitted electrodes indicate more than 0.05 mV since they have higher baseline noise than the textile-based dry electrodes. 

As shown in Figure 8b, the active signal of the reference electrode was 0.54 ± 0.05 mV. CS_1 and CS_2 show 0.58 ± 0.03 mV and 0.53 ± 0.04 mV, respectively. Therefore, those were a higher signal amplitude than the commercial electrode. In the case of embroidered and knitted electrodes, although it showed a relatively lower signal than the reference electrode, an EMG signal value of 0.30 mV or more was observed. Hence, the quality of the sEMG signal was evaluated by comparing the SNR value of the developed electrodes with those of the commercial electrode (Figure 8c). The SNR values of the reference, CS_1, CS_2, Em_LS, Em_MS, Kn_PL, Kn_PU, and Kn_IN were 31.10, 55.82, 68.28, 15.81, 15.85, 3.61, 4.73, and 7.03, respectively. On the other hand, when looking at the types of textile-based dry electrodes, the SNR values of the two types of electrodes made from conductive sheet were approximately twice that of the commercial electrodes. The entire electrode area acts as a contact area due to the excellent adhesion to the skin. In the case of Em_LS and Em_MS, the SNRs of similar values were shown, which was attributed to the difference in the embroidery shape of the 2D and 3D shapes depending on the manufacturing method. Em_MS confirmed that the area that can come into contact with the skin in 3D is wider than that of the Em_LS, but it has a similar result since the baseline noise increased due to movement. As mentioned earlier, the knit electrode exhibited a morphological change that was different from that of the conductive and embroidered electrode, that maintains the shape of the electrode when the sleeve is worn. Due to the shape of the deformed and expanded electrode, the noise was formed by the influence of adjacent muscles, and the muscle activity signal was reduced. 

Accordingly, since the reference electrode is a hydrogel-type electrode, it could be used in close contact with the skin, thus it was found to be stable against noise. Among the textile-type electrodes, the conductive sheet type produced less noise than the reference electrode, and it was confirmed that Em_LS and Em_MS showed similar levels. It was shown that textile-based dry electrode also possible to replace that wet electrode. In addition, during the muscle activation, CS_1 and CS_2 were able to acquire signal similarly to the reference electrode, and through structural improvement, through structural improvement, it was confirmed that textile based electrodes of embroidery and knitted type could be used as electromyography electrodes to replace Ag/AgCl electrodes.

**Figure 9:**
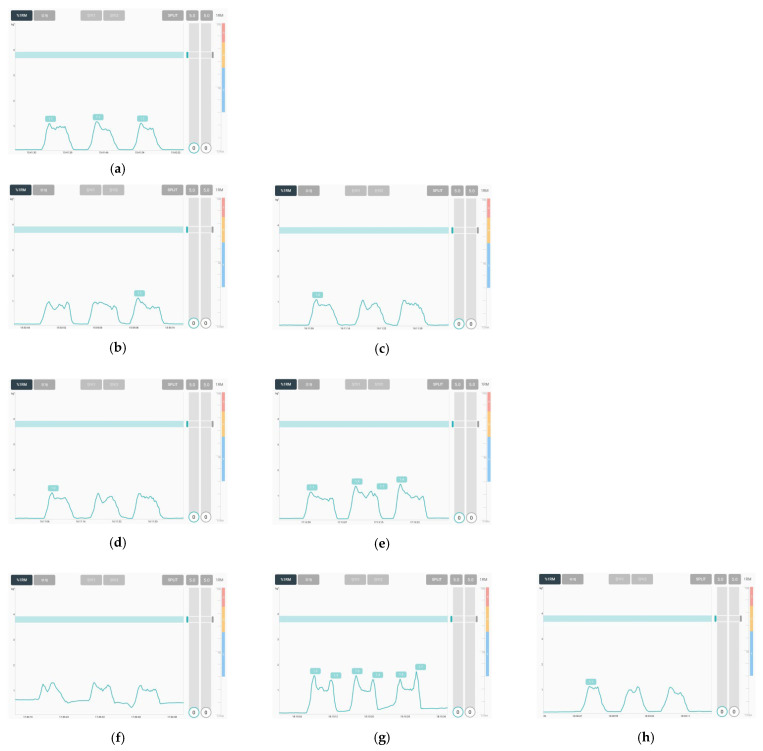
Images of collected sEMG signals by different types of embroidery-based textile electrodes with wearable devices. (**a**) Reference; (**b**) CS_1; (**c**) CS_2; (**d**) Em_LS; (**e**) EM_Ms; (**f**) Kn_PL; (**g**) Kn_PU; (**h**) Kn_IN.

In this study, to manufacture smart wear for measuring EMG signals in the future, the EMG signals were measured by combining a textile electrode with a commercially available wearable device, and the results are shown in Figure 9. The measurement method was carried out by connecting a commercial EMG wearable device to the electrode embedded in the leg sleeve in the same manner as the EMG signal acquisition method. Each sample was measured on the same scale. The developed electrodes were capable of obtaining an EMG signal. On the other hand, some different results were obtained in the same way as the results performed with the biopack. In the case of two types of conductive sheet-type electrodes and embroidery electrodes, the baseline noise was similar to that of the reference electrode, and the magnitude of the signal was also measured similarly. In the case of the knit electrode, however, the baseline noise of Kn_PU and IN was close to 0, but it was large in the case of Kn_PL. This was related to the shape of the electrode that changes after wearing according to the knit structure, similar to the one analyzed above. Accordingly, the EMG signal acquirement performance of the textile electrode was confirmed to see if it could be applied as a replaceable electrode for a commercial gel-type electrode. The optimal textile type can enable stable EMG signal acquirement by adjusting various parameters of the embroidery electrode and knit electrode later. Hence, dry electrodes can be developed.

## 4. Conclusions

Textile-based dry electrodes were fabricated under various manufacturing methods, and conditions, and electrical and EMG signal acquisition evaluations were conducted to confirm the possibility of replacing the previously used disposable electrodes and the applicability of textile-type dry electrodes.

The skin-impedance and EMG signal acquisition performance were different depending on the shape and structure of the textile-type dry electrode As a result of the analysis of the dry electrode sample, it was confirmed that as the skin-electrode adhesion was excellent, and the smaller the electrode shape and size change, the better the EMG signal collection performance. Accordingly, conductive sheet-type electrodes exhibited similar skin-electrode impedance to the conventional Ag/AgCl hydrogel electrode, noise amplitude at rest position, and signal amplitude at muscle activation state. The two types of embroidery electrodes (Em_LS and Em_MS) and three types of knit electrodes (Kn_PL, Kn_PU, and Kn_IN) made of conductive yarn exhibited higher skin-electrode impedance and noise amplitudes than the CS sample. The skin-electrode impedance of the knitted electrode was greater than that of the embroidered electrode. This was influenced by the change in the area of the electrode in contact with the skin due to the elasticity of the knit when wearing a knitted sleeve. The stretchability of the knit electrode deviated from the target muscle area and generated noise in EMG signal acquisition. On the other hand, in the case of the embroidery electrode, there was little change in shape, and thus the EMG signal acquisition performance was superior to that of the knit electrode. According to the embroidery technique, the Em_LS and Em_MS’s EMG signal amplitude was excellent in Em_LS, but the noise signal in Em_MS was smaller. Accordingly, similar values were observed in the SNR results, and the standard deviation was low in the case of Em_MS, confirming that it was more stable. Finally, when the EMG signal was acquired in connection with the wearable device, the signal could be acquired without noise except for the knit electrode.

In this study, various electrodes were fabricated using conductive sheets and conductive yarn, which are typical textile-type dry electrodes, and the possibility of replacing disposable Ag/AgCl hydrogel electrodes that are difficult to use for multiple uses and cause skin diseases when attached for a long time was confirmed. Accordingly, the conductive sheet-type electrode appeared most similar to the existing electrode. Moreover, although the sEMG signal acquirement of an embroidery or knit-based dry electrode was lower than Ag/AgCl hydrogel electrodes, it can be applied in the future by controlling the various parameters. Accordingly, by laminating or embedding the electrode on the fabric through three processes, it was confirmed that it can be used multiple times without considering the adhesion between the fabric and the electrode. It is expected that smart wear for collecting bio-signals that can collect signals similar to disposable electrodes can be manufactured by applying fiber/fabric-type dry electrodes in the future.

## Figures and Tables

**Table 1:** Sample code and images of the textile-based dry electrode used in this study.

Type	Sample Code	Designed Image	Sample Image
Conductive sheet	CS_1	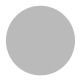	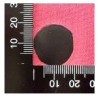
	CS_2		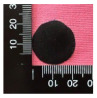
Embroidery	Em_LS	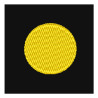	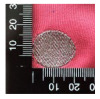
	Em_MS	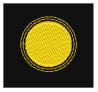	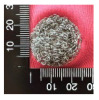
Knitting	Kn_PL	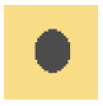 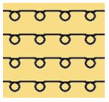	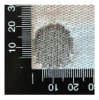
	Kn_PU	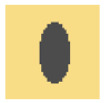 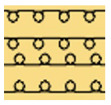	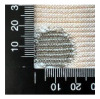
	Kn_IN	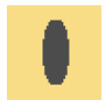 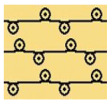	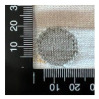

**Table 2:** Morphology and consumption of conductive yarn of the textile-based dry electrode.

Magnitude	Sample Code						
	Conductive Sheet		Embroidery		Knit		
	CS_1	CS_2	Em_LS	Em_MS	Kn_PL	Kn_PU	Kn_IN
Digital image	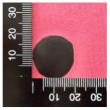	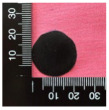	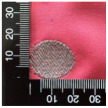	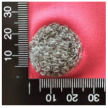	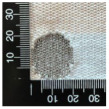	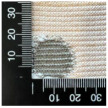	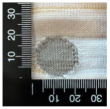
×35 (Front)	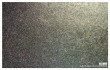	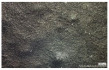	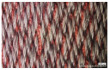	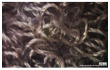	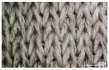	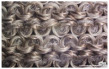	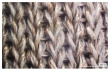
×35 (Back)	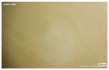	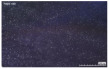	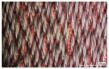	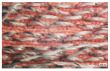	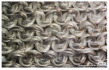	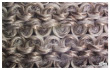	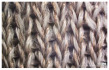
consumption of conductive yarn (cm)	-	-	624.0	878.4	643.5	756.5	724.5

## Data Availability

The data presented in this study are available in this article.

## Supplementary Materials

The following supporting information can be downloaded at: https://www.mdpi.com/article/10.3390/polym14173641/s1, Figure S1: Tensile properties of two types of conductive sheet.; Figure S2: 3D image of knitted electrode of plain structure (a) before and (b) after tensile test.

## References

[1] Guo L., Sandsjö L., Ortiz-Catalan M., Skrifvars M. (2020). Systematic review of textile-based electrodes for long-term and continuous surface electromyography recording. Text. Res. J..

[2] Niu X., Gao X., Liu Y., Liu H. (2021). Surface bioelectric dry electrodes: A review. Measurement.

[3] Kim H., Kim E., Choi C., Yeo W.-H. (2022). Advances in soft and dry electrodes for wearable health monitoring devices. Micromachines.

[4] Hassan M.M., Hossain M.M. (2021). Nanomaterials-patterned flexible electrodes for wearable health monitoring: A review. J. Mater. Sci..

[5] Shuvo I., Shah A., Dagdeviren C. (2022). Electronic textile sensors for decoding vital body signals: State-of-the-art review on characterization and recommendations. Adv. Intell. Syst..

[6] Kim S., Lee S., Jeong W. (2020). EMG measurement with textile-based electrodes in different electrodes sizes and clothing pressure for smart clothing design optimization. Polymers.

[7] Alizadeh-Meghrazi M., Ying B., Schlums A., Lam E., Eskandarian L., Abbas F., Sidhu G., Mahnam A., Moineau B., Popovic M.R. (2021). Evaluation of dry textile electrodes for long-term electrocardiographic monitoring. BioMed. Eng. OnLine.

[8] Lee S., Jamil B., Kim S., Choi Y. (2020). Fabric vest socket with embroidered electrodes for control of myoelectric prosthsis. Sensors.

[9] Spanu A., Botter A., Zedda A., Cerone G.L., Bonfiglio A., Pani D. (2021). Dynamic surface electromyography using stretchable screen-printed textile electrodes. IEEE Trans. Neural Syst. Rehabil. Eng..

[10] Togo S., Murai Y., Jiang Y., Yokoi H. (2019). Development of an sEMG sensor composed of two-layered conductive silicone with different carbon concentrations. Sci. Rep..

[11] Goncu-Herk G., Tuna B.G. (2021). The effect of sleeve pattern and fit on e-textile eleoctromyography (EMG) electrode performance in smart clothing design. Sensors.

[12] Kim H., Kim S., Lim D., Jeong W. (2022). Development and characterization of embroidery-based textile electrodes for surface EMG detection. Sensors.

[13] Lim D., Lee S., Roh S. (2022). Technical Embroidered E-Textile Products.

[14] Euler L., Guo L., Persson N.-K. (2021). Textile electrodes: Influence of knitting construction and pressure on the contact impedance. Sensors.

[15] Lam E., Alizadeh-Meghrazi M., Schlums A., Eskandarian L., Mahnam A., Moineau B., Popovic M.R. (2022). Exploring textile-based electrode materials for electromyography smart garments. J. Rehab. Ass. Technol. Eng..

[16] Stegeman D.F., Hermens H.J. Standards for Surface Electromyography: The European Project Surface EMG for Non-Invasive Assessment of Muscles (SENIAM). https://www.researchgate.net/publication/228486725.

[17] https://www.delsys.com/downloads/TUTORIAL/semg-detection-and-recording.pdf.

[18] Kim J.-H., Lee J.-S. (2014). Performance of breathable & waterproof jacquard fabric with PU-Nanofiber web and PU-film. Text. Sci. Eng..

[19] Murphy B.B., Scheid B.H., Hendricks Q., Apollo N.V., Litt B., Vitale F. (2021). Time evolution of the skin-electrode interface impedance under different skin treatments. Sensors.

[20] Barrera C.S., Piña-Martínez E., Roberts R., Rodriguez-Leal E. (2021). Impact of size and shape for textile surface electromyography electrodes: A study of the biceps brachii muscle. Text. Res. J..

