# Supplementary material for: Fabrication of Textile-Based Dry Electrode and Analysis of Its Surface EMG Signal for Applying Smart Wear
Source: Polymers (Basel). 2022 Sep 2;14(17):3641. doi: 10.3390/polym14173641 (PMC9460933; doi:10.3390/polym14173641)
Supplement: Supplementary file 1 [file polymers-14-03641-s001.zip › polymers-1842132-supplementary.pdf]

<Supplementary Materials>

Fabrication of Textile-Based Dry Electrode and Analysis of its Surface EMG Signal for Applying Smart Wear

Hyelim Kim[1], Soohyeon Rho [1,2], Sora Han[1], Daeyoung Lim,[1] and Wonyoung Jeong [1,*]

[1] Material and Component Convergence R&D Department, Korea Institute of Industrial Technology (KITECH), Ansan 15588, Gyeonggi-do, Korea

[2] Department of Nano Science and Technology, Sungkyunkwan University, Suwon 16419, Gyeonggi-do, Korea

* Correspondence: wyjeong@kitech.re.kr

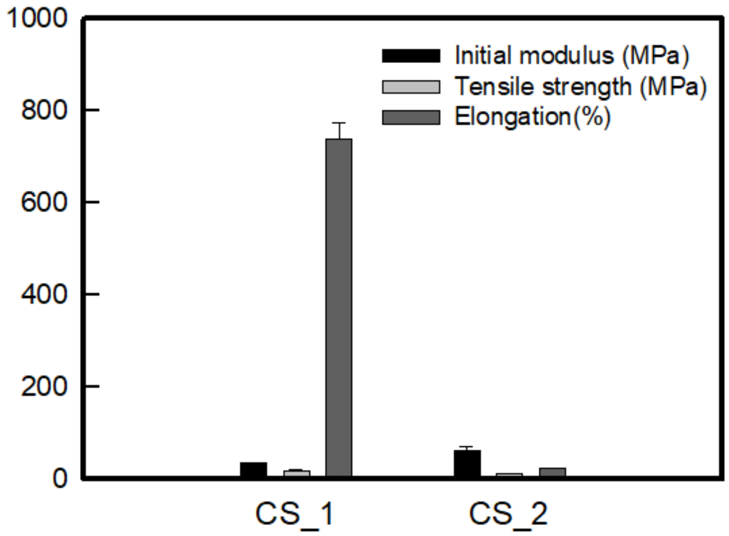

Figure S1. Tensile properties of two types of conductive sheet.

Figure S1 shows the tensile properties of two types of conductive sheets. The initial modulus of CS_1 was 34.5±1.0 MPa, the tensile strength was 19.5±2.7 MPa, and the elongation at break was 737.3±37.1 %. Initial modulus, tensile strength, and elongation at break of CS_2 are each 61.6±8.4 MPa, 12.8±0.6 MPa, and 23.3±0.4 %. Accordingly, while the initial modulus of CS_1 was lower than that of CS_2, the tensile strength and elongation at break were 1.5 times and 32 times higher, respectively. Accordingly, CS_1 was found to be a flexible yet strong material. As the flexability and elongation increases, the deformation occurs more easily, so in the case of CS_1, it is expected that it will affect the durability of the electrode part in smart wear, which is frequently worn on and off.

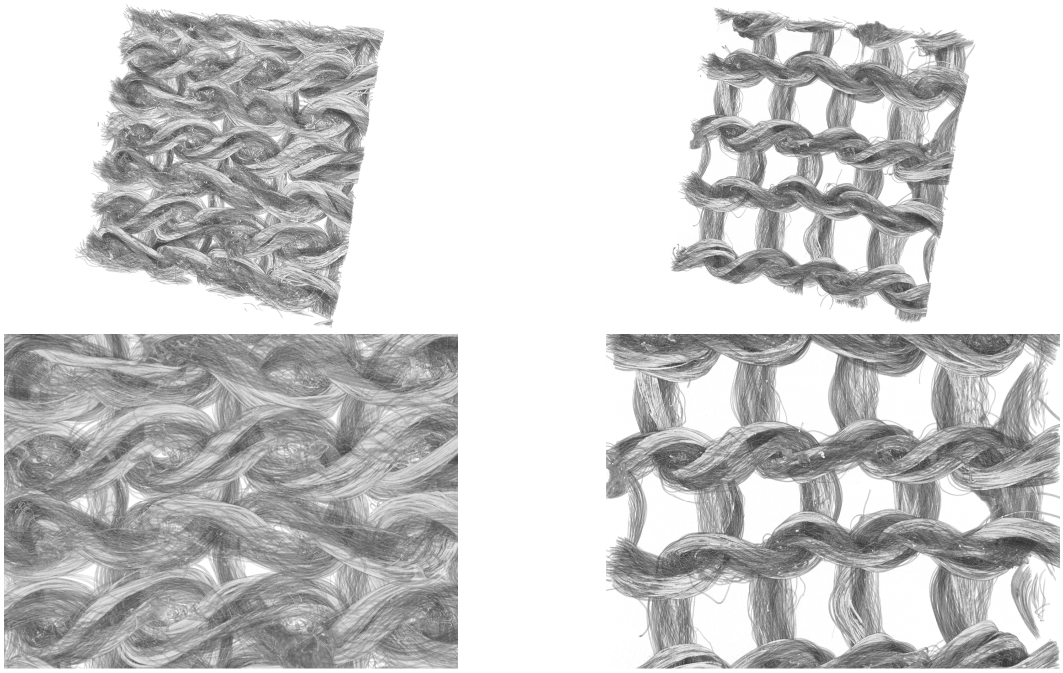

(a) Before tensile test (b) After tensile test

Figure S2. 3D image of knitted electrode of plain structure (a) before and (b) after tensile test.

Figure S2 confirms the structural change after 80% elongated using X-ray microscopy to confirm the morphological change of the knitted electrode of plain structure that varies before and after wearing. At this time, three loops were selected and analyzed to compare the change in loop length. The loop before tension was 2.21 mm and 1.44 mm in the wale and course directions, respectively. After tensioning, the loops were 2.18 mm and 1.76 mm in each direction. Accordingly, the strains in the wale and course directions are -1.18% and 18.1%, respectively. As the sample increases by about 20% in the transverse direction, it is expected that the EMG signal collection will be affected.
